# Incidence and predictors of postoperative complications in Sub-Saharan Africa: a systematic review and meta-analysis

**DOI:** 10.3389/frhs.2024.1353788

**Published:** 2024-05-09

**Authors:** Daniel Aboma Yadeta, Tsegahun Manyazewal, Dereje Bayissa Demessie, Dyre Kleive

**Affiliations:** ^1^ School of Public Health, St. Paul’s Hospital Millennium Medical College, Addis Ababa, Ethiopia; ^2^ College of Health Sciences, Addis Ababa University, Addis Ababa, Ethiopia; ^3^ Oslo University Hospital, Oslo, Norway

**Keywords:** quality measure, essential surgery, postoperative complications, meta-analysis, Sub-Saharan Africa

## Abstract

**Background:**

Postoperative complications remain a significant challenge, especially in settings where healthcare access and infrastructure disparities exacerbate. This systematic review and meta-analysis aimed to determine the pooled incidence and risk factors of postoperative complications among patients undergoing essential surgery in Sub-Saharan Africa (SSA).

**Method:**

PubMed/MEDLINE, EMBASE, CINAHL, Web of Science, and Google Scholar were searched from January 2010 to November 2022 for completed studies reporting the incidence and risk factors associated with postoperative complications among patients undergoing essential surgery in SSA. Severity of postoperative complications was ranked based on the Clavien-Dindo classification system, while risk factors were classified into three groups based on the Donabedian structure-process-outcome quality evaluation framework. Studies quality was appraised using the JBI Meta-Analysis of Statistics Assessment and Review Instrument (JBI-MAStARI), and data were analyzed using Comprehensive Meta-Analysis (CMA) software. The study protocol adhered to the PRISMA guidelines and was registered in PROSPERO (CRD42023414342).

**Results:**

The meta-analysis included 19 studies (10 cohort and 9 cross-sectional) comprising a total of 24,136 patients. The pooled incidence of postoperative complications in SSA was 20.2% (95% CI: 18.7%–21.8%), with a substantial heterogeneity of incidence observed. The incidence varied from 14.6% to 27.5% based on the Clavien-Dindo classification. The random-effects model indicated significant heterogeneity among the studies (*Q* = 54.202, *I* = 66.791%, *p* < 0.001). Contributing factors to postoperative complications were: structure-related factors, which included the availability and accessibility of resources, as well as the quality of both the surgical facility and the hospital.; process-related factors, which encompassed surgical skills, adherence to protocols, evidence-based practices, and the quality of postoperative care; and patient outcome-related factors such as age, comorbidities, alcohol use, and overall patient health status.

**Conclusion:**

The meta-analysis reveals a high frequency of postoperative complications in SSA, with noticeable discrepancies among the studies. The analysis highlights a range of factors, encompassing structural, procedural, and patient outcome-related aspects, that contribute to these complications. The findings underscore the necessity for targeted interventions aimed at reducing complications and improving the overall quality of surgical care in the region.

**Systematic Reviews Registration:**

https://www.crd.york.ac.uk/PROSPERO/, identifier (CRD42023414342).

## Introduction

Surgery is an essential aspect of healthcare globally, playing a significant role in preventing, diagnosing and treating various medical conditions (1). Emergency and essential surgical care, according to the World Health Organization, refers to the provision of surgical services that are crucial for addressing life-threatening conditions, preventing disability, and improving overall health outcomes in a community or population (2). However, postoperative complications remain a significant challenge, especially in Sub-Saharan Africa (SSA) where healthcare access and infrastructure disparities exacerbate (3).

Quality of surgical care is crucial for optimal patient outcomes. Quality of care refers to healthcare services that meet patient needs and expectations while achieving desired health outcomes (4). The Donabedian quality model examines healthcare quality using three elements: structure, process, and outcome (5). To provide appropriate care, it is necessary to have sufficient access to staff, equipment, and facilities. Research has shown that a shortage of these resources is linked to a higher incidence of postoperative complications (6, 7). A high-quality process involves promptly recognizing and managing complications, and utilizing the best techniques to minimize them (8). A high-quality outcome in the context of healthcare, particularly in surgery, refers to achieving the best possible results for the patient following a procedure or treatment (9). By evaluating these elements, the Donabedian model assesses the quality of care for post-operative complications, reflecting the overall quality of care offered.

Post-operative complications refer to adverse events or outcomes that occur as a result of a surgical procedure. Surgical complications can encompass a wide range of issues, including infections, bleeding, organ damage, adverse reactions to anesthesia, wound complications (such as dehiscence or hernias), blood clots, and surgical errors (10).

Surgical patients in sub-Saharan Africa (SSA) confront formidable hurdles stemming from deficient healthcare facilities, scarce resources, inadequate infrastructure, and insufficient professional training, all of which elevate the risk of postoperative complications and exacerbate the burden of surgical diseases in the region (11). However, comprehensively understanding these challenges is impeded by the lack of standardization in data collection and reporting, as well as by variations in study populations and settings. This inconsistency hinders accurate assessment of complication prevalence and severity, complicating efforts to address these issues effectively (12).

Therefore, a comprehensive synthesis of the available literature is necessary to identify common patterns and risk factors for postoperative complications in SSA. This systematic review and meta-analysis aimed to provide a comprehensive overview of the aggregated incidence and risk factors of postoperative complications among surgical patients in SSA.

## Methods

### Study design

The protocol for this systematic review and meta-analysis has been registered at the International Prospective Register of Systematic Reviews (PROSPERO) database, ID: CRD42023414342, and adhered to the PRISMA guidelines for the design and reporting of the results.

### Search strategy

PubMed/MEDLINE, EMBASE, CINAHL, Web of Science, and Google Scholar were searched from January 2010 to November 2022 for completed studies that reported the incidence and risk factors of postoperative complications among patients undergoing Emergency & essential surgery in SSA. The year 2010 marked a pivotal period where significant attention was drawn to the burden of surgical disease in sub-Saharan Africa, as highlighted in existing literature (13). Additional studies were searched manually from reference lists of some important articles. Controlled medical subject headings (MeSHs) terms and keywords words were used in different combinations using Boolean Operators. The keywords included surgery, postoperative, incidence, risk, and sub-Saharan Africa.

### Eligibility criteria

PICOS (participants, interventions, comparison, outcomes, and study designs) design was used to establish the eligibility criteria.

- Participants: Patients of any age in SSA undergoing essential surgery.
- Intervention: Emergency and Essential surgery, which was referred to, based on the WHO guidelines (11), as a set of surgical procedures that are considered crucial for addressing substantial health needs.
- Comparison: Articles with or without a comparator were eligible.
- Outcomes: Primary outcome: incidence of postoperative complications, with the severity of the surgical complications ranked based on the Clavien Dindo classification system (14). Secondary outcome: risk factors for postoperative complications which are categorized into three groups based on the Donabedian structure-process-outcome framework for evaluation of quality of healthcare and services.
- Study design: No restrictions on study designs.

The classification of outcomes was conducted by the authors of the manuscript during the study's methodology and data analysis phases.

Studies were excluded if done outside SSA, carried out in animal models, not reported in the English language, or were non-empirical publications such as reviews, editorials, commentaries, or conference abstracts.

### Study selection

Two independent authors screened the titles and abstracts of identified studies based on selection criteria and using a standardized form that guided their evaluation process. Studies that were duplicates or did not meet the inclusion criteria in the initial title and abstract searches were excluded and full texts of the remaining studies were further evaluated. Any disagreements between the authors were resolved through discussions. Mendeley Desktop Version 1.19.8 software was used to control potential duplicates.

### Data extraction

The Joanna Briggs Institute Meta-Analysis Of Statistics Assessment And Review Instrument (JBI-MAStARI) was used to extract descriptive data from the included articles. The data extracted include surname of the first author, year of publication, country, study population, sample size, data collection method(s), outcome measures, data analysis, and any study limitations reported by the author.

### Assessment of risk of bias

Studies that matched the inclusion criteria were appraised using the JBI System for the Unified Management, Assessment, and Review of Information (JBI-SUMARI) tool (15). The JBI-MAStARI was used to evaluate studies with quantitative evidence. The evaluation was conducted by two independent reviewers. The appraisal tool had nine risk of bias questions that the reviewers used to score each article as low (0–3), moderate (4–6), or high quality (15).

Heterogeneity was evaluated using standard statistical tests (chi-square and *I*^2^) and subgroup analysis if statistical pooling was not feasible.

### Statistical analysis

Comprehensive Meta-Analysis (CMA) software was used to do the meta-analysis. Effect sizes were expressed as event rates for categorical data with a 95% confidence interval (CI). The study's outcomes of interest were measured as categorical or continuous variables, and odds ratios or regression coefficients were collected, along with data on potential confounding factors.

## Results

### Characteristics of included studies

**Figure 1:**
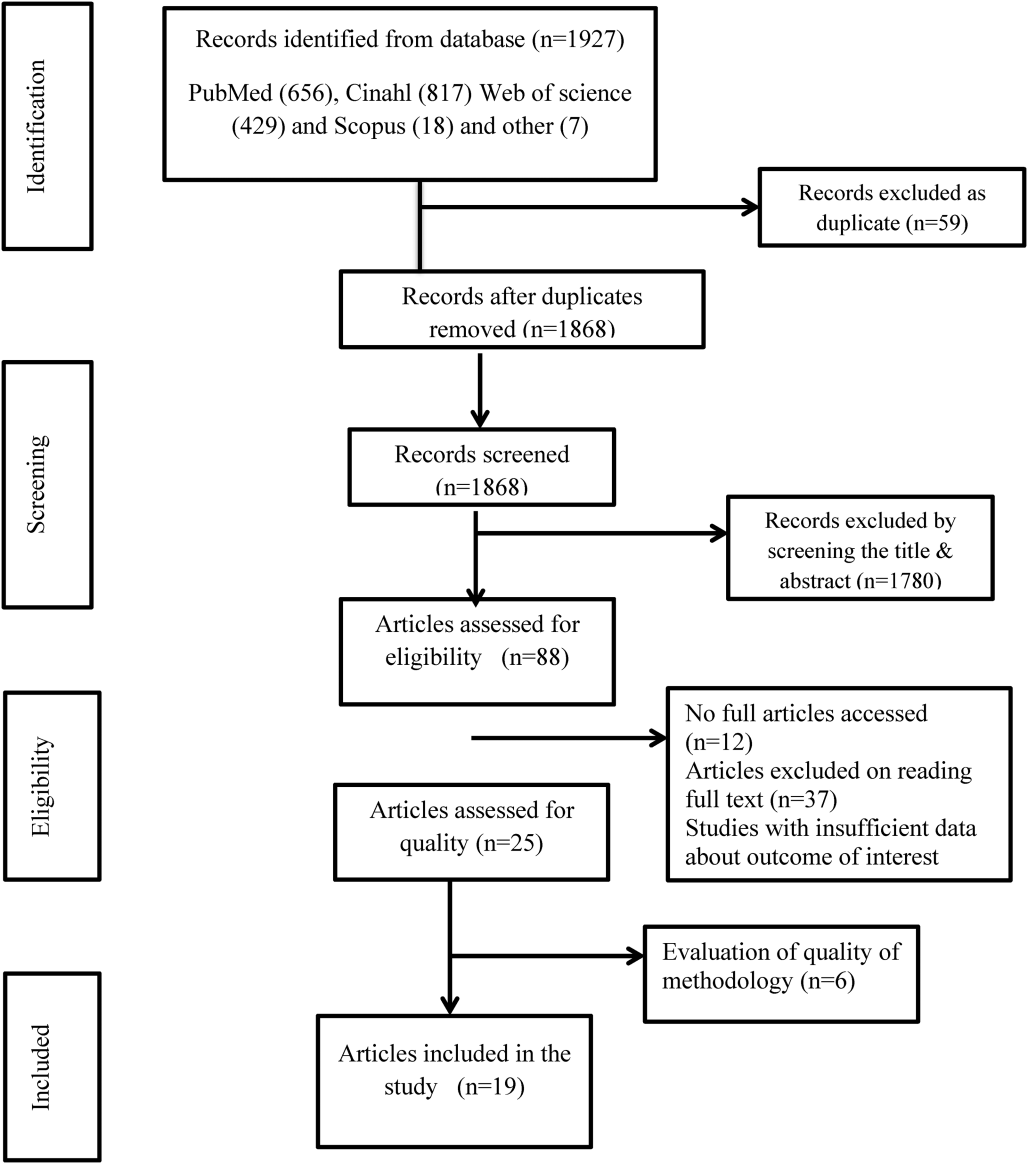
PRISMA flow diagram of the study.

A total of 1,927 potentially relevant records were identified in the initial search of the databases, of which 1,868 remained after removing 59 duplicates. After title and abstract screening, 1,764 studies were excluded and the remaining 104 articles were analyzed in full-text, of which 19 met the inclusion criteria and were included in the final analysis (Figure 1).

The table summarizes the characteristics of the 19 articles included in a meta-analysis conducted across various countries in sub-Saharan Africa between 2007 and 2021. Out of the 19 included articles, nine were cross-sectional studies, with three of the nine rated as high quality. Nine of the studies used systematic random sampling to select participants, while the remaining articles followed a cohort study design, with five out of ten rated as high quality. The samples taken were representative, and outcomes were measured using structured questionnaires. The outcomes focused on postoperative complications, mortality, and surgical site infections across neurosurgery, trauma surgery, and abdominal surgery. All studies controlled for confounding factors and employed various statistical tests for data analysis. Limitations identified included potential selection bias, poor follow-up, and generalizability concerns, highlighting the challenges and considerations in the studies (Table 1).

**Table 1:** Characteristics of the studies included in the review.

No	1st author, year of publication	Aim(s) and study design & type of hospital	Country & year of study	Population & sample size	Data collection method (s), & tools	Outcome measure & type of surgery	Measures of quality & data analysis	Limitations identified by the author(s)	Quality score
1.	Abaver et al. (16)	Determine etiology and incidence of hospital-acquired infections and their associated risk factors following neurosurgical procedures; multi-center, cross-sectional	South Africa; October 2013–September 2014	All inpatients who had had a neurosurgical procedure at & *1,688	Electronic data extraction tool	Surgical site infection & neurology	Chi-square	Not stated	M
2.	Botchey et al. (17)	Quantify the burden of injuries and patient outcomes; prospective cohort, single center	Kenya; January 2014–June 2015	Patients presenting at the emergency department with at least 1 injury & *8,701	Paper based Structured questionnaire based on International Classification of Diseases codes	In-hospital death & Trauma	Bivariate and multivariate logistic regressions	Potential selection bias due to single hospital-based trauma registry; low utilization of formal medical services likely underestimates the number of injured patients; prehospital deaths likely missed due to transportation	M
3.	Derseh et al. (18)	Assess results after surgery for intestinal obstruction; hospital-based, multi-center, cross-sectional	Ethiopia; 01 January 2014–31 December 2017	All patients surgically treated for intestinal obstruction *254	Structured data abstraction sheet (from medical chart)	Postoperative complication(Dehiscence, SSI, Pneumonia, Shock and Death	Bivariate LR & MVLR	Not stated	M
4.	Grema et al. (19)	Provide an overview of the spectrum of typhoid ileal perforation cases and their outcome; cross-sectional, single center	Nigeria; January–December 2016	All patients admitted with typhoid ileal perforation & *471	Data abstraction sheet from chart	Postoperative complication, gastro intestinal	Not specified; Fisher's exact test	Generalizability	M
5.	Henry et al. (20)	Investigate the association between elevated serum lactate and outcomes following major abdominal surgery; cohort, single center	Uganda; March–November 2020	All patients admitted for major abdominal surgery & *246	Structured paper based data abstraction form	In hospital mortality, gastero intestinal	Not specified; logistic regression	Random error at the time of sampling	H
6.	Hernandez et al. (21)	Externally validate grading system in patients with appendicitis; multicenter, cohort	South Africa; 2010–2016	Patients with acute appendicitis & *1,415 patients	Semi-structured questionnaire	Post-operative complication, gastero intestinal	Mortality and POC; Uni and Multivariate analysis	Generalizability to LMIC population	H
7.	Legesse et al. (22)	Identify risk factors associated with in-hospital postoperative complications; multi-center, cohort	Ethiopia; 27 May–22 August 2017	Pediatric patients undergoing surgery & *2,048	Pretested paper based data collection tool	Incidence of in-hospital postoperative complications, gastero intestinal	Bivariate LR,MVLR,X2 or Fisher's exact tests, *t*-test or Manne_Whitney *U*-test	Not stated	H
8.	Mawalla et al. (23)	Establish the prevalence, pattern, and predictors of surgical site infection; cross-sectional, single center	Tanzania; July 2009–March 2010	All patients who underwent major surgery in surgical wards & *265	Paper based standardized data abstraction form	Incidence of surgical site infection, gastero-Intestinal	BVLR & MVLR	Low participation rate, poor follow-up	M
9.	Onen et al. (24)	Evaluate performance of SAS in predicting outcomes in patients undergoing laparotomy; single-center & cohort	Uganda; January–April 2021	Adult patients undergoing laparotomy & *151	Structured data abstraction sheet (from medical chart) & telephone	Post-operative complication, gastero intestinal	Bivariate LR & MVLR	Over-estimation of study variable	H
10.	Muchuweti and Jönsson (7)	Prospectively determine frequency and risk factors for abdominal SSIs; multi-center, cohort	Zimbabwe; May 2007–June 2008	All patients above 15 years undergoing elective or emergency abdominal operations & *285	Interviewer-administered structured questionnaire (telephone)	Incidence of SSI & Gastero Intestinal	Bivariate LR & MVLR	Not stated	M
11.	Osinaike et al. (25)	Determine post-operative complications, critical care admissions, and mortality following elective surgery; multi-center, cohort	Nigeria; July 9–16 2018	Admitted patients undergoing elective surgery with a planned overnight hospital stay following surgery & *1,425	Paper case record form	In-hospital postoperative complications & mortality	Bivariate LR & MVLR	Not stated	M
12	Torborg et al. (26)	Identify risk factors associated with in-hospital postoperative complication; multi-center, cohort	South Africa; 22 May–22 August 2017	Pediatrics patients (aged <16yrs) undergoing surgery & *2,048	Interviewer-administered structured questionnaire	Incidence of in-hospital postoperative complication, gastro intestinal	Bivariate LR MVLR,X2 or Fisher's exact tests, *t*-test or Manne_Whitney *U*-test		H
13.	Mohamed et al. (27)	Investigate the time interval from emergency department presentation to TBI management interventions for patients presenting with TBI; single-center, cross-sectional	Uganda; 2016–2017	All patients presenting to the emergency department with suspected or documented TBI & 3,944	Data abstraction sheet from chart and Semi-structured questionnaire	Time of first intervention delivery & neurology	Pearson's *χ*^2^, &logistic regression	Selection bias, generalizability	H
14.	Ntudu et al. (28)	Determine the relationships among the causes, characteristics, patterns and outcomes of abdominal injury patients undergoing operations; cohort	Tanzania; August 2016–August 2017	All patients undergoing operations with a diagnosis of abdominal trauma & *210	Electronic (ODK) Pre-tested questionnaire	Outcome of surgical intervention, gastero intestinal	Bivariable analysis Multivariable analysis.	Selective bias, &small sample size	H
15.	Weldu et al. (11)	Assess prevalence and associated factors of surgical site infections; cross-sectional	Ethiopia; 02 February–31 March 2016	All post-operative patients & *281	Interviewer-administered Piloted questionnaire (Papeer form)	Surgical site infection	Bivariable analysis; Multivariable analysis.	Single hospital generalizability Recall bias and social desirability bias	H
16	Mangi et al. (29)	to identify risk factors associated with in-hospital postoperative complication & multi-center cohort study design	South Africa & Jan 22, 2017, and Jun 22, 2019	Pediatrics patients undergoing surgery & *432	Face to face interview	incidence of in-hospital postoperative complication, gastero Intestinal	Bivariate LR MVLR	Prone to recall bias	H
17	Sincavage et al. (9)	Identify risk factors associated with in-hospital postoperative complication in patients with appendicitis; multicenter, cohort	Tanzania; July–September 2016	Patients with acute appendicitis & *734 patients	Data abstraction sheet from chart and Semi-structured questionnaire	Post-operative complication, gastero intestinal	Mortality and POC, Univariate and Multi analysis	Generalizability	H
18.	Laeke et al. (30)	Assess the pattern of CS rates according to the Robson classification and describe maternal and perinatal outcomes; multicenter, ohort	Ethiopia; 02 September–31 March 2018	All post-operative patients & *381	Interviewer-administered structured questionnaire	Surgical site infection	Bivariable analysis Multivariable analysis.	Single hospital, generalizability, &recall and social desirability biases	H
19.	Kintu et al. (31)	Assess results after surgery for intestinal obstruction in a hospital; multi-center, cross-sectional	Mali; 01 January 2014–31 December 2019	All patients surgically treated for intestinal obstruction *354	Paper based Data (Sociodemographic and clinical) abstraction sheet from chart	Postoperative complication, gastero intestinal	Bivariate LR & MVLR	Not stated	M

* Represents the sample size of the included article.

POC, post-operative complication.

The records of studies excluded in the full-text reviews with underlying reasons are summarized in Supplementary File S1. Findings from methodological quality assessment of the cross-sectional and cohort studies included in the meta-analysis are summarized in Table 2.

**Table 2:** Methodological quality assessment of cross-sectional and cohort studies.

First author, year of publication	Q1	Q2	Q3	Q4	Q5	Q6	Q7	Q8	Q9	Q10	Q11	Overall quality of the study
Cross-sectional studies												
Weldu et al. (11)	N	Y	Y	Y	Y	Y	Y	Y				7/8 (High)
Abaver et al. (16)	N	Y	Y	Y	U	N	Y	Y				5/8 (Moderate)
Derseh et al. (18)	N	Y	Y	Y	Y	Y	Y	Y				7/8 (High)
Grema et al. (19)	Y	Y	Y	Y	Y	N	Y	Y				7/8 (High)
Mawalla et al. (23)	Y	Y	Y	Y	U	N	N	Y				6/8 (Moderate)
Mangi et al. (29)	N	Y	Y	N	Y	N	Y	Y				6/8 (Moderate)
Sincavage et al. (9)	Y	Y	Y	Y	U	N	N	Y				6/8 (Moderate)
Osinaike et al. (25)	Y	N	Y	Y	N	Y	Y	Y				6/8 (Moderate)
Mohamed et al. (27)	N	Y	Y	Y	N	Y	Y	Y				6/8 (Moderate)
# studies achieved compliance	4	7	8	7	4	4	7	9				
Cohort studies												
Botchey et al. (17)	N	Y	Y	Y	Y	N	Y	Y	Y	Y	Y	9/11 (High)
Henry et al. (20)	N	Y	Y	Y	Y	Y	Y	Y	Y	Y	Y	9/11 (High)
Hernandez et al. (21)	Y	Y	Y	Y	Y	Y	Y	Y	N	Y	Y	7/11 (Moderate)
Legesse et al. (22)	N	Y	Y	Y	N	Y	Y	Y	N	Y	Y	7/11 (Moderate)
Muchuweti and Jönsson (7)	Y	Y	Y	Y	Y	N	Y	Y	N	Y	Y	7/11 (Moderate)
Torborg et al. (26)	N	Y	N	Y	Y	N	Y	Y	N	Y	Y	7/11 (Moderate)
Ntudu et al. (28)	Y	Y	N	Y	N	Y	Y	Y	N	Y	Y	7/11 (Moderate)
Kintu et al. (31)	N	Y	Y	Y	Y	Y	Y	Y	Y	Y	Y	9/11 (High)
Onen et al. (24)	Y	Y	N	Y	N	Y	Y	Y	N	Y	Y	7/11 (Moderate)
Laeke et al. (30)	Y	N	Y	Y	Y	Y	Y	Y	Y	Y	Y	9/11 (High)
# studies achieved compliance	5	7	7	10	7	7	10	10	4	10	10	

Criteria were adapted from the JBI Critical Appraisal Checklist for descriptive/case series research. For Cross sectional studies: (1) was the study based on a random or pseudo-random sample? (2) Were the criteria for inclusion in the sample clearly defined? (3) Were confounding factors identified and strategies to deal with them stated? (4) Were outcomes assessed using objective criteria? (5) If comparisons were being made, was there sufficient description of the groups? (6) Were the outcomes of people who withdrew described and included in the analysis? (7) Were outcomes measured in a reliable way? (8) Was appropriate statistical analysis used? High quality: meets ≥7 criteria, Moderate quality: meets ≥4 criteria, Low quality: <4 criteria.

**For Cohort studies:** (1) were the two groups similar and recruited from the same population? (2) Were the exposures measured similarly to assign people to both exposed and unexposed groups? (3) Was the exposure measured in a valid and reliable way? (4) Were confounding factors identified? (5) Were strategies to deal with confounding factors stated? (6) were the groups/participants free of the outcome at the start of the study (or at the moment of exposure)? (7) Were the outcomes measured in a valid and reliable way? (8) Was the follow up time reported and sufficient to be long enough for outcomes to occur? (9) Was follow up complete, and if not, were the reasons to loss to follow up described and explored? (10) Were strategies to address incomplete follow up utilized? (11) Was appropriate statistical analysis used? Each item was rated Y = Yes, N = No or U = Unclear. Unclear was awarded where not enough information was provided. Cut-off points for determining the quality of the study are as follows:—Low quality: Score of 0–3—Moderate quality: Score of 4–6 and High quality: Score of 7–9.

### Incidence of postoperative complications

**Figure 2:**
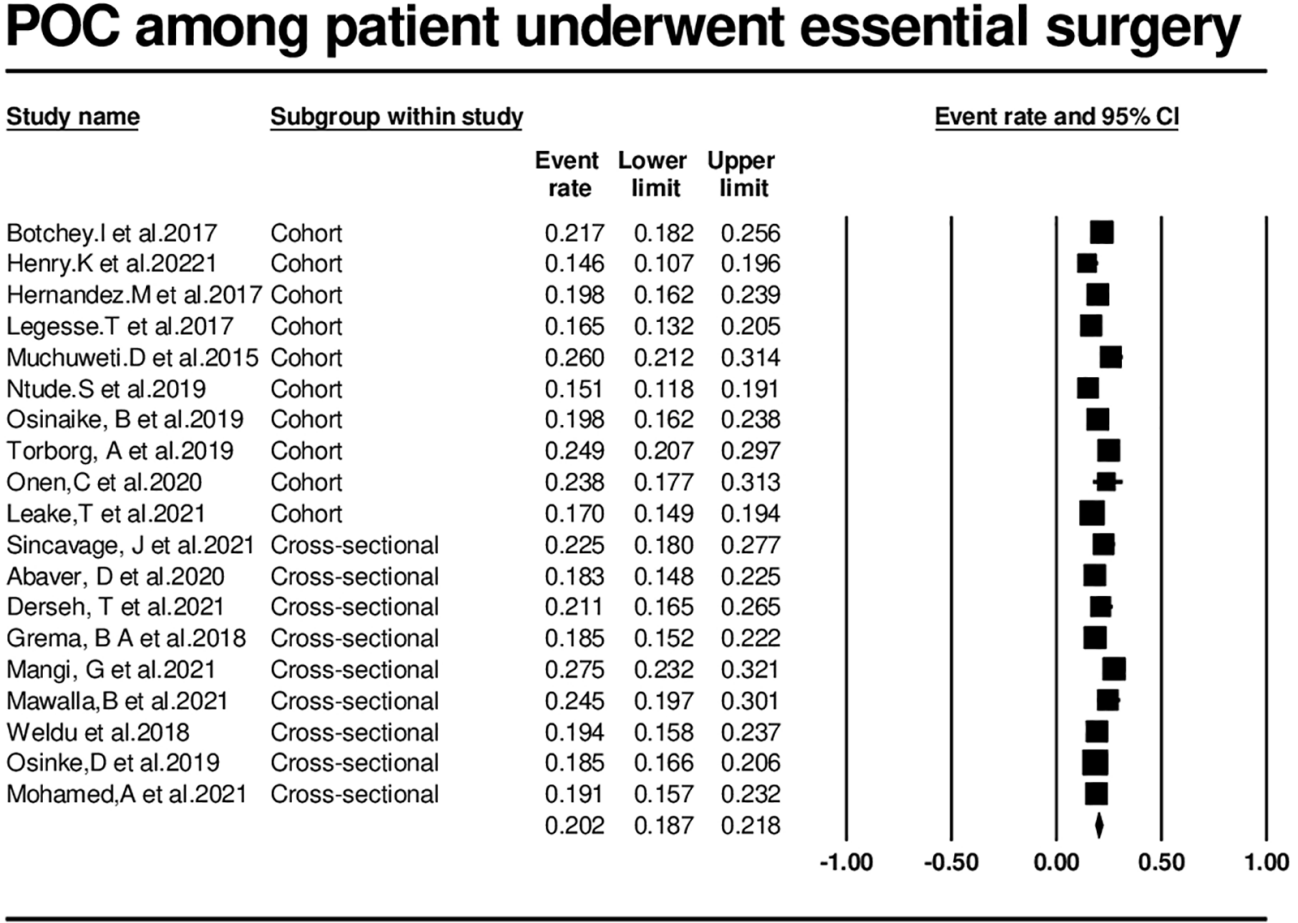
Forest plot of the incidence of postoperative complications among surgical patients.

In the meta-analysis of the nineteen studies, a total of 24,136 patients were included, with 2,372 experiencing postoperative complications after undergoing essential surgery. The overall incidence of postoperative complications was calculated to be 20.2% (95% CI: 18.7%–21.8%) using the random-effects model, showing significant heterogeneity among the studies. The incidence ranged from 14.6% to 27.5% based on the Clavein-Dindo classification system (Figure 2).

### Donabedian quality measures

Nineteen studies utilized the Donabedian quality model to evaluate healthcare quality using the three dimensions of structure, process, and outcome across diverse settings. Among 19 studies, fourteen (73.6%) evaluated the structure and process, 12 (80%) evaluated Process relate factor seventeen (89.5%) and nineteen (100%) articles were Outcome related factors evaluated the process. The studies focus on a range of topics, including surgical site infections, postoperative complications, mortality, risk factors for poor outcomes, and predictors of in-hospital death.

The identified factors have been categorized into three groups—structure, process, and outcomes—based on the Donabedian framework for the evaluation of the quality of healthcare and services. Overview of the statistically significant factors identified in the studies (Table 3).

**Table 3:** Summary of the statistically significant risk factors of postoperative complications in Sub-saharan Africa.

First Author, year of publication	Factors contributing to postoperative complications		
	Structure-related factors: Healthcare system factors, including availability and accessibility of resources, staffing, quality of surgical facility	Process-related factors: Factors related to the surgical process itself, including surgical skill, use of evidence-based practices, adherence to established protocols, quality of postoperative care	Patient outcome-related factors: Patient's overall health status, comorbidities, age, and other individual characteristics that may impact the outcome of the surgery
Abaver et al. (16)		Prolonged stay in the hospital (>30 days)	Patient age
Botchey et al. (17)	Mechanisim of injury & prehospital care	Use of drain; Use of iodine alone in skin preparation; Duration of operation ≥3 h,	Presence of pre-morbid illness(Cigarette smoking & longer prehospital times, and severe injury severity scores
Derseh et al. (18)	Admission path (from emergecy deprt,) & rocess of diagnosis and initial management duration of illness, and preoperative diagnoses	Preoperative diagnosis Gangrenous small bowel &	Age group of ≥55 & Duration of illness of ≥24
Grema et al. (19)	Need for ICU admission & availablity of emergency case operating room separately	Type of surgery (Emergency laparotomy)	High-SAS category
Henry et al. (20)	Pre-surgical antibiotics & avialblity of safety protocol	Duration between diagnosis & surgery for emergencies (days); Longer than a day; Duration of surgery >1.5 h	Age >18 years & Presence of Comorbidities
Hernandez et al. (21)	Reduced access to advanced imaging techniques	inability of power prognostic clinical decision making tools (Alvarado score) & Greater than 3 days of preoperative symptoms	late presentation of patient & presence of peritonitis at admission
Legesse et al. (22)	Preoperative hospital stays more than 7 days	Duration of operation more than 1 h; Administering antimicrobial prophylaxis before 1 h of operation	Patients charactersitcs (Age, Sex) & Presence of comorbidities
Mawalla et al. (23)	Availablity Essential surgical equipments and supplies	Use of drain; Use of iodine alone in skin preparation; Duration of operation ≥3 h	Presence of pre-morbid illness (Cigarette smoking
Onen et al. (24)	Interrupted or poor referral linkage between health facilities	delays in making diagnosis and surgical intervention & Anesthesia related	Patients charactersitcs (Age, Sex) & High SAS classification, Presence of comorbidities
Torborg et al. (26)	Turn over of trained manpower and hospital infrastructure (beds, OR light and table)	Urgency of surgery Routine; Severity of surgery (major) &Identification of risk factors for perioperative complications	ASA physical status; Infective indication for surgery
Mohamed et al. (27)	Availability of CT-scan & staffing trained manpower	Duration of operation >1.5 h	Patients charactersitcs (Age, Sex) & Presence of comorbidities
Ntudu et al. (28)	Mode of transport to hospital & Mechanism of injury		Severe injury on the NISS
Weldu et al. (11)	Post-operative hospital stays from 8 to 14 days	Use of local anesthesia; Dirty incision classification	Patients charactersitcs (Age, Sex) & Presence of comorbidities & History of alcohol use
Osinaike et al. (25)		Emergency surgery & surgical checklist uses	Age of patient >35 years & pre-existing comorbidity
Muchuweti and Jönsson (7)	Length of hospital stay Duration & Time of detection of SSI and type of bacteria & Length of operation Duration	Urgency of surgery Routine; Severity of surgery (major) & prophylactic antibiotics	ASA physical status II
Sincavage et al. (9)		Postoperative disposition &Postoperative length of stay	Patients charactersitcs (Age, Sex) & Presence of comorbidities & ASA physical status classification
Laeke et al. (30)	deficits within both prehospital and hospital care	length of hospital stay	Age, and admission GCS score,
Mangi et al. (29)	Interruption of miniblood bank		Demographic characteristics (age,) & Pre-operative anaemia
Traut et al. (34)		Duration of operation & adherance to safety checklist	Patients charactersitcs (Age, Sex) & Presence of comorbidities

ICU, intensive care uni; CT, computerized tomograph; SAS, Surgical Apgrar Scor; ASA, American Society of anesthesiologist; NISS, new injury severity score.

### Structure-related factors

Among the nineteen studies, seventeen clearly have pinpointed structural factors that influence surgical procedures. The identified factors encompass various aspects of both prehospital and hospital care (25). These include the mechanism of injury, Patient admission path(direct from emergency department to Operating room or Surgical ward/unit), and the process of diagnosis and initial management (17). Factors like the duration of illness, preoperative diagnoses, and the need for ICU admission were also highlighted (19) Moreover, the availability of emergency case operating rooms, pre-surgical antibiotics, and essential surgical equipment were crucial considerations (20, 29). Issues such as reduced access to advanced imaging techniques, interrupted referral linkages between health facilities, and turnover of trained manpower contribute to deficits in care. Additionally, factors like staffing of trained manpower, mode of transport to the hospital, and the length of post-operative hospital stays further impact patient outcomes. Detection time of surgical site infections, types of bacteria involved, and the duration of operations also play significant roles in determining outcomes (22, 32).

### Process-related factors

Among the nineteen studies, seventeen clearly delineate process-related factors influencing surgical outcomes. Prolonged hospital stays exceeding 30 days and the implementation of specific procedures such as drains and iodine skin preparation emerge as prevalent risk factors for postoperative complications (16, 33). Factors like preoperative diagnosis of gangrenous small bowel, emergency laparotomy, and extended time between diagnosis and surgical intervention were identified as contributors to adverse outcomes (21). Additionally, the administration of antimicrobial prophylaxis within one hour of operation was recognized as a significant risk factor, Urgency and severity of surgery, operation duration surpassing 1.5 h, the use of local anesthesia, and dirty incision classification further underscore the complexity of adverse process-related outcomes (34).

### Patient outcome-related factors

All included studies have highlighted different risk factors influencing surgical outcomes and leading to postoperative complications (16, 18). Notably, patient age has emerged as a common factor, with individuals aged 35 years or older, at higher risk of complications (23) Additionally, pre-existing illnesses and comorbidities significantly contribute to adverse effects (23, 28). Factors such as smoking and a history of alcohol use was linked to increased postoperative complication risks (30). Other significant contributors include the presence of peritonitis upon admission, pre-anesthesia medical comorbidities classified by the ASA Physical Status Classification System, and severe injury defined by the New Injury Severity Score (26), Moreover, the use of drains during surgery and iodine alone in skin preparation was associated with elevated complication risks following abdominal surgery (16, 33).

### Heterogeneity

The random-effects model indicated significant heterogeneity among the studies (*Q*-value = 54.202, *p* < 0.001, *I*-squared = 66.791%), demonstrating that the variation in effect sizes was not purely random. The Tau-squared value of 0.029 indicated a substantial degree of heterogeneity among the studies.

### Subgroup analysis

**Figure 3:**
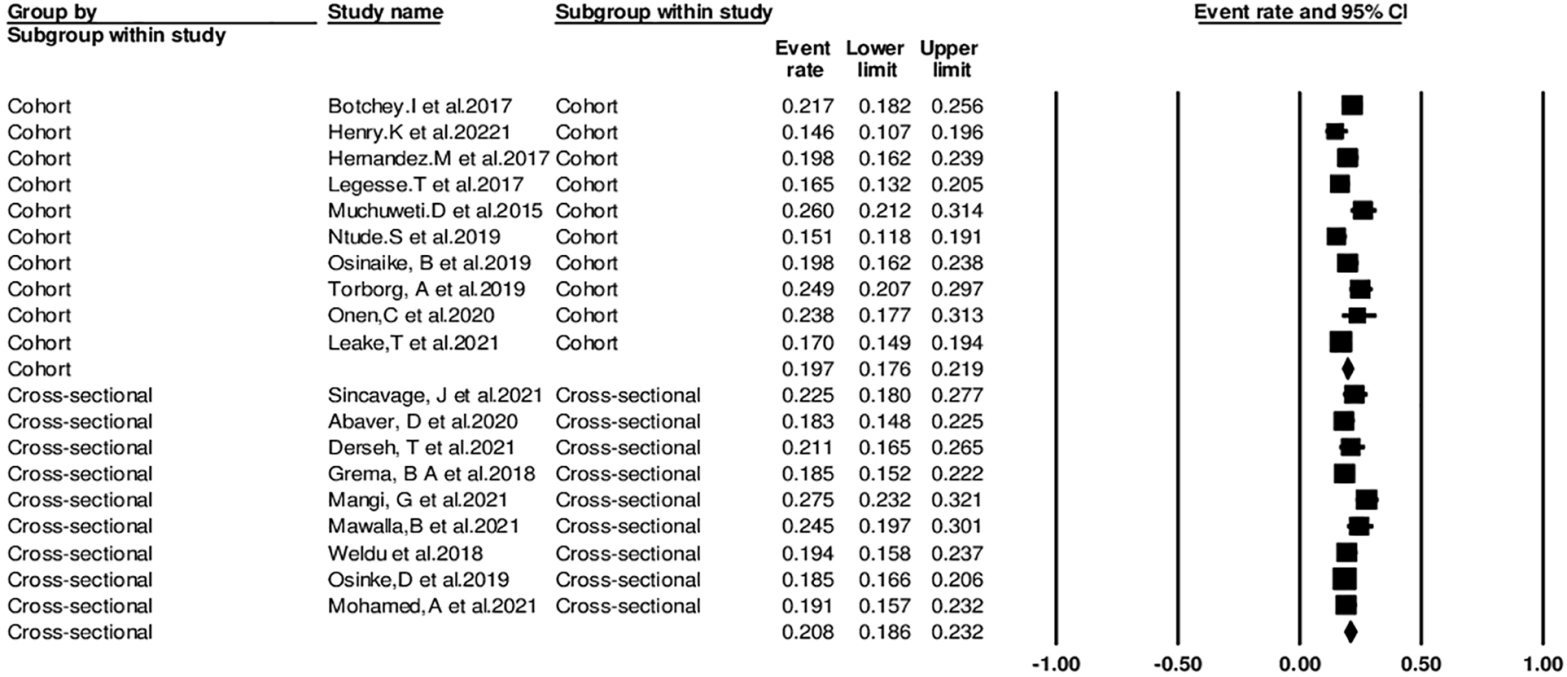
Subgroup analysis based on study design.

The pooled incidence of postoperative complications based on the Clavein-Dindo classification system in the seven cross-sectional studies was 20.8% (95% CI: 18.6%–23.2%), while the pooled incidence in the remaining cohort studies was 19.7% (95% CI: 17.6%–21.9%). The difference in incidence between the two study designs was statistically significant (*p = 0.001*). Therefore, the type of study design appears to be a significant source of heterogeneity (Figure 3).

### Sensitivity analysis

The results showed that no individual study significantly affected the overall incidence estimate of post-operative complications by more than 1%, indicating that our results were robust and not driven by a single study.

### Publication bias

**Figure 4:**
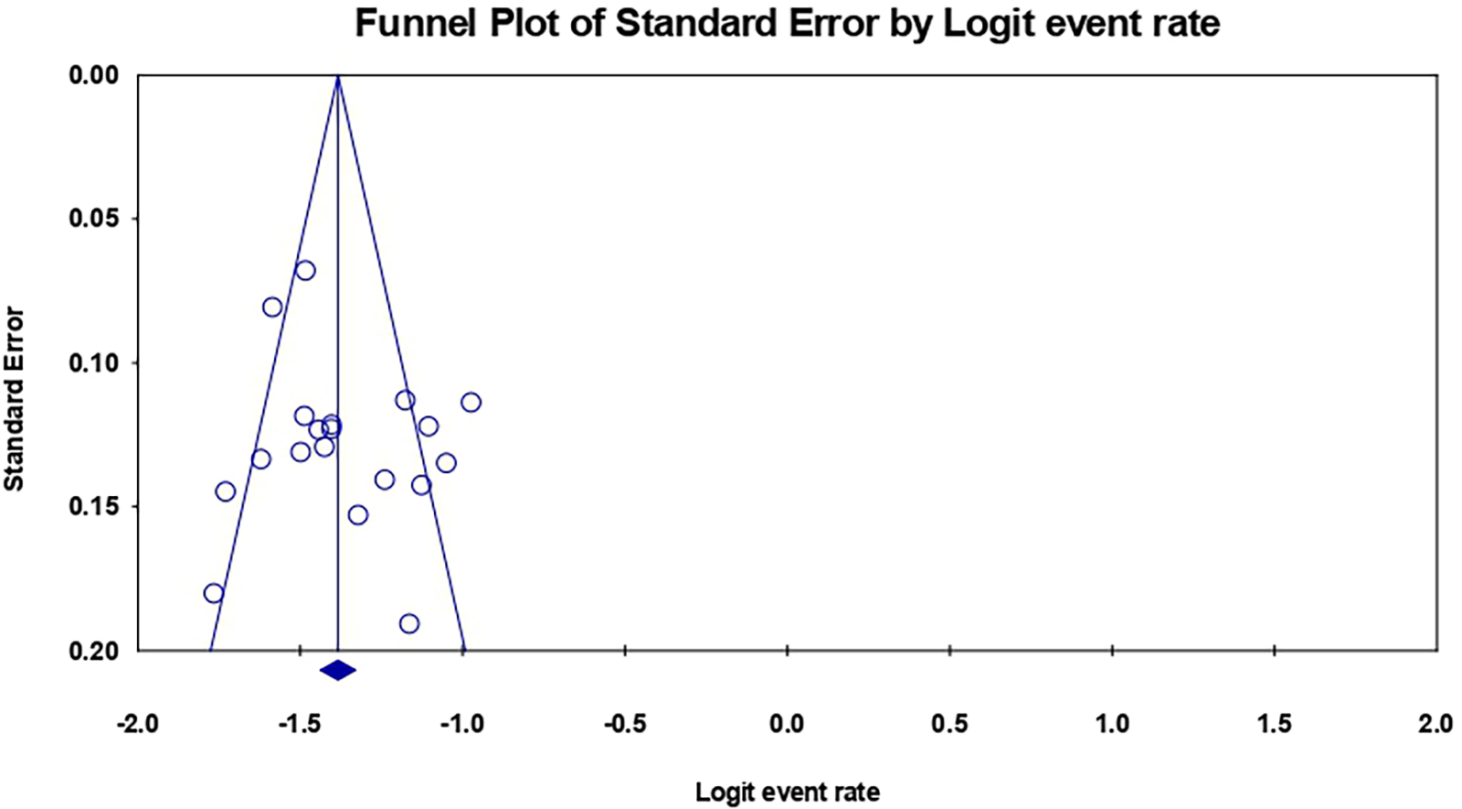
Funnel plot of the included studies in this meta-analysis for the incidence of postoperative complications.

The funnel plot revealed asymmetry pinpointed to the left (Figure 4), suggesting a potential publication bias in the included studies. To further investigate this, Egger's test was conducted, yielding a significant result (*p < 0.339*), providing additional evidence for the absence of publication bias.

## Discussion

Postoperative complications are adverse events that occur after surgery and can significantly impact a patient's recovery and outcome. According to this meta-analysis, these complications may be influenced by patient-related factors such as age, pre-morbid illness, smoking, alcohol use, and severity of injury, as well as process-related factors such as duration of operation, use of drains, skin preparation, antimicrobial prophylaxis, and type of surgery.

### Structure-related factors

Structure-related factors within healthcare systems play a pivotal role in shaping surgical outcomes, encompassing various elements such as resource availability, staffing levels, and the quality of surgical facilities. Our analysis underscores the significant impact of these factors on patient care and the overall success of surgical interventions.

The mechanism of injury and pre-hospital care set the stage for subsequent treatment outcomes. Adequate pre-hospital care, including timely assessment and stabilization of patients, is crucial in optimizing outcomes and minimizing the risk of complications upon hospital admission (35, 36). However, deficits in pre-hospital care, such as delays in transport or inadequate emergency medical services, can impede timely access to surgical intervention and exacerbate patient outcomes (37).

The admission path from the emergency department to the operating room or surgical unit is another critical determinant of surgical outcomes. Efficient processes for triage, diagnosis, and initial management are essential in expediting care delivery and facilitating prompt surgical intervention when indicated (38). However, interruptions or delays in this pathway can prolong the time to surgery and increase the risk of adverse outcomes (38).

The availability of resources, including access to intensive care units (ICUs) and emergency case operating rooms is paramount in ensuring timely and appropriate surgical care. Adequate staffing levels and the presence of trained manpower are essential for delivering high-quality surgical services and responding effectively to surgical emergencies (39). Similarly, the availability of essential surgical equipment and supplies is vital in facilitating safe and efficient surgical procedures (9).

Challenges such as interrupted or poor referral linkages between health facilities can hinder access to specialized care and delay surgical intervention, particularly in rural or underserved areas (40, 41). Moreover, high turnover rates of trained manpower and inadequate hospital infrastructure pose significant challenges to maintaining consistent surgical services and may contribute to variations in care quality (42, 43).

Access to advanced imaging techniques, such as computed tomography (CT) scans, is essential for accurate preoperative evaluation and surgical planning (44). However, reduced access to these resources may limit diagnostic capabilities and hinder the timely identification of surgical conditions, potentially leading to delayed or suboptimal treatment.

Postoperative care, including the duration of hospital stays and the detection of surgical site infections (SSIs), also reflects structural factors within healthcare systems (44). Prolonged hospital stays may indicate underlying issues such as inadequate postoperative care or challenges in discharge planning. Similarly, delays in SSI detection may stem from deficiencies in infection control measures or limited access to diagnostic resources (45).

### Process-related factors

Process-related factors play a critical role in determining surgical outcomes, encompassing various aspects of the surgical process itself. Factors such as surgical skill, adherence to established protocols, and the use of evidence-based practices are fundamental in ensuring the success of surgical interventions (46, 47). However, our analysis highlights several specific process-related factors that significantly impact postoperative complications.

Prolonged hospital stays exceeding 30 days emerged as a notable risk factor for adverse outcomes. Extended hospitalization not only increases the risk of nosocomial infections but also reflects underlying systemic issues in healthcare delivery, such as delayed discharge planning and inadequate postoperative care (46, 47).

The use of drains and iodine alone in skin preparation during surgery has also been associated with increased postoperative complications. While drains are often employed to prevent fluid accumulation and facilitate wound healing, their indiscriminate use may introduce the risk of infection and other complications (48, 49). Similarly, the use of iodine alone in skin preparation, rather than more comprehensive preoperative skin antisepsis methods, may predispose patients to surgical site infections (50).

Furthermore, the duration of the operation emerged as a significant determinant of postoperative complications. Operations lasting more than three hours pose inherent challenges, including prolonged exposure to anaesthesia and increased surgical stress, which can heighten the risk of adverse outcomes (50).

Preoperative factors, such as the diagnosis of gangrenous small bowel and the necessity for emergency laparotomy, also contribute to adverse surgical outcomes. These conditions often require urgent surgical intervention, leaving little time for thorough preoperative optimization and increasing the complexity of the procedure, thereby elevating the risk of complications (50).

Additionally, delays in making diagnoses and interventions, particularly in emergency settings, exacerbate the risk of adverse outcomes. Prompt recognition and timely intervention are crucial in mitigating the progression of surgical conditions and preventing complications associated with delayed treatment (51).

Anesthesia-related factors, such as the choice of anesthesia and adherence to safety protocols, also influence surgical outcomes. Local anesthesia may offer advantages in certain procedures but must be carefully selected based on patient factors and procedural requirements to minimize complications (52).

The urgency and severity of surgery, as well as the use of prophylactic antibiotics, are further determinants of postoperative complications. Routine surgeries may carry lower inherent risks compared to major or emergency procedures, while the timely administration of prophylactic antibiotics is essential in preventing surgical site infections and reducing the overall risk of complications (53).

Postoperative disposition and length of hospital stay also impact patient outcomes. Efficient postoperative care and discharge planning are crucial in facilitating patient recovery and reducing the risk of complications associated with prolonged hospitalization (54).

#### Patient outcome-related factors

Patient outcome-related factors play a crucial role in determining the success of surgical interventions, encompassing various individual characteristics such as overall health status, comorbidities, age, and other demographic factors (55). Our analysis highlights the significance of these factors in predicting surgical outcomes and guiding patient management strategies.

Advanced age has consistently emerged as a significant predictor of surgical outcomes, with individuals aged 35 years and above being at higher risk of adverse events. The presence of pre-morbid illnesses, including factors such as cigarette smoking, longer pre-hospital times, and severe injury severity scores, further compounds the risk of postoperative complications (56).

Patients with comorbidities, such as pre-existing medical conditions or a high severity of illness as indicated by the High-SAS category, are particularly vulnerable to adverse surgical outcomes (57). Additionally, late presentation of patients, especially those with symptoms of peritonitis upon admission, poses challenges in timely intervention and may exacerbate postoperative morbidity and mortality rates (58).

Demographic characteristics, including age and sex, interact with the presence of comorbidities to influence surgical outcomes. Notably, older patients with pre-existing comorbidities are at heightened risk, underscoring the importance of comprehensive preoperative evaluation and risk stratification in this population (10).

The American Society of Anesthesiologists (ASA) physical status classification system provides valuable insights into patients' overall health status and perioperative risk, with higher ASA classifications correlating with increased complication rates (10). Similarly, the New Injury Severity Score (NISS) serves as a predictor of postoperative outcomes, reflecting the severity of traumatic injuries and guiding treatment decisions (10).

Other patient-related factors, such as a history of alcohol use, pre-operative anemia, and admission Glasgow Coma Scale (GCS) score, further contribute to the complexity of surgical risk assessment (10). Understanding these factors and their interplay is essential for tailoring treatment plans and optimizing patient outcomes.

## Conclusion

Our meta-analysis highlights the prevalence of postoperative complications affecting 20.2% of essential surgery procedures in Sub-Saharan countries.

Structural factors significantly influence surgical outcomes and patient care delivery. Addressing challenges related to resource availability, staffing, infrastructure, and care coordination is essential to optimize surgical services and improve patient outcomes. By investing in robust healthcare systems and implementing strategies to overcome barriers, policymakers and healthcare providers can enhance the quality and accessibility of surgical care.

Recognizing and addressing process-related factors are crucial for optimizing surgical outcomes. Prioritizing evidence-based practices, adhering to established protocols, and implementing comprehensive perioperative care strategies can effectively minimize the risk of postoperative complications and enhance patient safety and satisfaction.

Patient outcome-related factors play a pivotal role in shaping surgical outcomes and should be meticulously considered in preoperative assessment and perioperative management. By identifying high-risk patients, implementing evidence-based interventions, and fostering multidisciplinary collaboration, healthcare providers can mitigate the impact of these factors and elevate the overall quality of surgical care.

## Data Availability

The original contributions presented in the study are included in the article/**Supplementary Material**, further inquiries can be directed to the corresponding author.
