# Supplementary material for: Incidence and predictors of postoperative complications in Sub-Saharan Africa: a systematic review and meta-analysis
Source: Front Health Serv. 2024 May 9;4:1353788. doi: 10.3389/frhs.2024.1353788 (PMC11112115; doi:10.3389/frhs.2024.1353788)
Supplement: Supplementary file 1 [file Table1.docx]

**Supplementary Table 1:** Records excluded at full text examinations with reasons

| **No** | **Author** | **Title** | **Reason for exclusion** |
| --- | --- | --- | --- |
| 1 | Maroyi.R et al | Characteristics and Outcomes of Surgical Patients with Solid Cancers Admitted to the Intensive Care Unit. | It assessed the aim of the study was to describe the components of the Mobile Surgical Outreach (MSO) program and its results as a model of care delivery for women with genital fistula. It did not identify the potential factors affecting the quality of surgical care |
| 2 | Higuchi.S et al | Incidence and complications of perioperative atrial fibrillation after non-cardiac surgery for malignancy | The study aimed to illustrate the clinical impact of perioperative atrial fibrillation (POAF) on mortality and morbidity in major non-cardiac surgery for malignancy. Therefore, surgery for malignancy is not a category of DC3-priority. |
| 3 | Nyangena.E et al | Profile of care given to patients with blunt chest injuries within the first 48 hours | The study aimed at describing the nature of care that patients with blunt chest injuries received during the first 48 hours after injury; it does not assess factors that affect quality of surgical car |
| 4 | Rushton.A et al | Development and validation of two clinical prediction models to inform clinical decision-making for lumbar spinal fusion surgery for degenerative disorders and rehabilitation following surgery: protocol for a prospective observational study | The aim of this study is to develop a clinical prediction model to predict which patients are likely to have favorable outcomes following lumbar spinal fusion surgery (LSFS) and to predict which patients are likely to have favorable long-term outcomes to inform rehabilitation, it is not about Determinants of quality of surgical care |
| 5 | Barnes.R et al. 2018 | Preoperative education in hip and knee arthroplasty patients in Bloemfontein. | The aim of the study was to determine the educational needs of patients who underwent elective total hip or knee arthroplasty (THA or TKA) in private hospitals not focusing on quality of surgical care |
| 6 | Esquivel.M et al. 2016 | Mapping Disparities in Access to Safe, Timely, and Essential Surgical Care in Zambia | The aim is to analyze the infrastructure, capacity, and availability of surgical care in Zambia to inform health policy priorities. It does not focus on the on quality of surgical care rather it is about access for service |
| 7 | Ogbuanya.A et al.2022 | Mortality audit in general surgery unit and lessons learned at a Nigerian tertiary hospital: a single-center observational study | The aim was to document the pattern and factors that influence mortality in the general surgery unit of our institution. Therefore, it is not focusing on the quality of surgical care service |
| 8 | Van Waes.O et al.2013 | Management of penetrating injuries of the upper extremities | The study is aimed to assess the validity selective non operative management in penetrating upper extremities trauma in a tertiary referral trauma center; therefore the study does not focus on the quality of surgical care |
| 9 | Antosh.D et al.2017 | Feasibility of prophylactic salpingectomy during vaginal hysterectomy | The primary objective of this study was to determine the feasibility of bilateral salpingectomy at the time of vaginal hysterectomy. Secondary objectives included identification of factors associated with unsuccessful salpingectomy and assessment of its impact on operating time, blood loss, surgical complications, and menopausal symptoms; the study is not published yet |
| 10 | Chale.G et al.2021 | Clinical indications for total abdominal hysterectomy among women seen in Dar es Salaam regional referral hospitals, Tanzania: a prospective, observational hospital-based study | To assess the clinical indications among women undergoing total abdominal hysterectomy; therefore, this is not quality study on surgical care |
| 11 | Burgess.J et al.2021 | Adverse events during anesthesia at an Ethiopian referral hospital: a prospective observational study. | To investigate the incidence and types of adverse event during anesthesia occurring in a major referral, hospital in Ethiopia, not quality of surgical care |
| 12 | Bhangu.A et al.2019 | Global variation in anastomosis and end colostomy formation following left-sided colorectal resection. | The study aims to assess global variation in end colostomy rates after left-sided colorectal resection. It is a comparative cohort study of middle- or high-income countries with low-income countries |
| 13 | Concepcion.T et al.2019 | Prevalence of Pediatric Surgical Conditions Across Somaliland | The aim of the study is to estimate the national burden of surgical disease among children in Somaliland using a nationwide community-based household survey it is not quality of surgical care |
| 14 | Singaram.S et al.2020 | The physical impact of long bone fractures on adults in KwaZulu-Natal | The study aims to investigate the physical impact of long bone fractures on adults; therefore, this study mainly focusses on the impact of long bone fracture on daily activities of individuals, it is not a study focusing on the factors affecting quality of surgical care |
| 15 | Gallaher.J et al. 2021 | Underutilization of Operative Capacity at the District Hospital Level in a Resource-Limited Setting | The study aims to describe the potential underutilization of operative capacity at the district hospital for patients needing an exploratory laparotomy, it is not a study about surgical outcome |
| 17 | Davidson.B et al.2019 | Outcomes and challenges of a kidney transplant program at Groote Schuur Hospital, Cape Town: A South African perspective. | The study is aimed to determine patient and graft survival estimates and factors associated with poor outcomes in kidney transplant recipients not about quality of surgical care |
| 19 | Chokotho.L et al. 2020 | A review of existing trauma and musculoskeletal impairment (TMSI) care capacity in East, Central, and Southern Africa | The study is aimed to compare patient-reported quality of life and functional status after femoral shaft fractures treated with IMN or ST. it is more focusing on quality of life but not about quality of surgical care |
| 20 | Masumbuko.C et al.2019 | Delayed surgery leads to reduced elbow range of motion in children with supracondylar humeral fractures managed at a referral hospital in sub-Saharan Africa | The study is aimed to evaluate the effect of delays in the surgical management of pediatric SHFs treatment on the range of motion ROM at the elbow at 12 weeks follow-up |
| 21 | David.B et al.2019 | Nigerian surgical outcomes – Report of a 7-day prospective cohort study and external validation of the African surgical outcomes study surgical risk calculator | The study aimed to contribute to data on post-operative complications, critical care admissions and mortality following elective surgery |
| 23 | Krige.J et al.2014 | The management of complex pancreatic injuries | The study is aimed to evaluate the outcome after a pancreatoduodenectomy for non-reconstruct able pancreatic injuries. The |
| 24 | Tognon.F et al.2019 | Analysis of caesarean section and neonatal outcome using the Robson classification in a rural district hospital in Tanzania: an observational retrospective study. | The study is aimed to assess the pattern of CS rates according to the Robson classification and describe maternal and perinatal outcomes |
| 25 | Ali.A et al.2022 | The effect of ethnicity on the incidence of postoperative nausea and vomiting in moderate to high-risk patients undergoing general anesthesia in south Africa: a controlled observational study. | The study is aimed to explore the wide range of clinical presentations, associated risk factors, complications, and surgical management of perforated peptic ulcer patients &; it does not focus on the quality of surgical outcome |
| 26 | Jaferson.B et al.2022 | Surgical Apgar score as a predictor of outcomes in patients following laparotomy at Mulago National Referral Hospital, Uganda: a prospective cohort study | The aim is to determine the performance of SAS in predicting complications and mortality in patients undergoing laparotomy |
| 27 | Temesgen.F et al.2021 | Early Outcome of Laparotomy Wounds in Pediatric Patients in TASH, Addis Ababa, Ethiopia: Six-Months Prospective Study. | Is study of prevalence and risk factors of SSI and wound dehiscence in children undergoing laparotomy in the pediatric surgery |
| 28 | Lekuya.H et al.2018 | Patient satisfaction with peri-operative anesthesia care and associated factors at two National Referral Hospitals: a cross sectional study in Eritrea | Is study focusing on evaluating the short-term outcomes of patients with degloving injuries in the surgical units |
| 29 | Adeleye.A et al.2018 | Determinants of the in-hospital outcome of spontaneous itch in a developing country in the ct era: A neurosurgical prospective analytical study | The aim of the study is to determine the incidence, risk factors, and outcome of surgical site infections |
| 30 | Francisca et al. 2016 | Determinants of surgical outcomes of small intestinal atresia at Kenyatta national hospital, a dissertation submitted as part fulfillment for the award of master of medicine in pediatrics surgery , | The study is aimed to determine the factors that affect surgical outcomes of small intestinal atresia |
| 31 | Andemeskel et al.2019 | Patient satisfaction with peri-operative anesthesia care and associated factors at two National Referral Hospitals: a cross sectional study in Eritrea | The objective of study is of assess the level of patients’ satisfaction with anesthesia service, the reason for not including |
| 32 | Beniami.D et al.2015 | Abdominal surgical site infections: a prospective study of determinant factors in Harare, Zimbabwe | The study is aimed To estimate the prevalence of pediatric surgical conditions : therefore this is not a study of quality rather it is a prevalence study |
| 33 | Amponsah.G et al.2017 | Patient satisfaction with peri-operative anesthesia care and associated factors at two National Referral Hospitals: a cross sectional study in Eritrea. | To determine the prevalence of PA among non-cardiac surgical patients and its implications for their postoperative outcomes, this is a study of prevalence |
| 34 | Haas.R et al.2020 | Prospective Study of Surgery for Traumatic Brain Injury in Addis Ababa, Ethiopia: Surgical Procedures, Complications, and Postoperative Outcomes | to evaluate the effectiveness and safety of perioperative radiotherapy (RT) in patients with solitary fibrous tumor |
| 35 | Suresh.L et al.2022 | The epidemiology and outcomes of prolonged trauma care (EpiC) study: methodology of a prospective multicenter observational study in the Western Cape of South Africa. | The study is aims to investigate how the delivery of resuscitative interventions and their timeliness impacts the morbidity and mortality outcomes of patients with critical injuries |
| 36 | Mathieu.L et al.2014 | Early management of traumatic brain injury in a Tertiary hospital in Central Kenya: A clinical audit | To describe the epidemiological characteristics and outcomes of patients with open fractures |
| 37 | Hu.J et al.2016 | Predicting postoperative opioid use with machine learning and insurance claims in opioid-naïve patients | to evaluate the safety of total thyroidectomy for thyroid disorders and summarize the treatment experience in a less-developed region |
